# Circulating oligodendrocyte-derived extracellular vesicle surface markers in athletes exposed to repetitive head impacts: an exploratory study

**DOI:** 10.3389/fneur.2026.1915481

**Published:** 2026-09-24

**Authors:** Masato Mitsuhashi, Haiping Sun, Li Xing, Akihiro Hirata, Yuko Oguma, Hiroyuki Ishida, Keisuke Kawata, Viviana Jimenez, Vernon B. Williams

**Affiliations:** 1 NanoSomiX Inc., Irvine, CA, United States; 2 Irvine Materials Research Institute, University of California, Irvine, Irvine, CA, United States; 3 Sports Medicine Research Center, Keio University, Yokohama City, Kanagawa, Japan; 4 School of Public Health, Indiana University, Bloomington, IN, United States; 5 Center for Sports Neurology & Pain Medicine at Cedars-Sinai Kerlan-Jobe Institute, Los Angeles, CA, United States; 6 California State Athletic Commission, Sacramento, CA, United States

**Keywords:** axonal injury, blood biomarkers, concussion, extracellular vesicles, neuroinflammation, oligodendrocytes, repetitive head impacts, subconcussion

## Abstract

**Background and objectives:**

Repetitive head impacts (RHI) are increasingly recognized as potential contributors to long-term neurological consequences. Current clinical assessments rely primarily on symptoms and acute neurological findings, limiting the ability to detect and longitudinally monitor biological responses that may occur in the absence of clinically recognized concussion. This exploratory study investigated surface-associated biomarkers on circulating oligodendrocyte-derived extracellular vesicles (ODEs) as candidate blood markers of biological responses associated with RHI exposure.

**Methods:**

Plasma ODEs were captured using an anti-myelin oligodendrocyte glycoprotein (MOG) antibody and probed for ciliary neurotrophic factor (CNTF), interleukin-1β (IL-1β), and α-synuclein (α-syn), selected to reflect neural repair, neuroinflammatory, and neurodegenerative processes, respectively. Unlike conventional EV analyses focused primarily on intravesicular cargo, this platform preserves vesicle integrity and quantifies biomolecules associated with the external surface of captured ODEs. Assays for each biomarker were analytically characterized prior to application to the athletic cohorts.

**Results:**

Longitudinal measurements were obtained from boxing and mixed martial arts fighters (*n* = 18; pre-event to 5 weeks post-event), high school American football players (*n* = 34; sampled monthly from July through November), and college rugby players (*n* = 14; sampled 3–4 times annually from 2014 to 2017). High school cross-country runners (*n* = 68) served as an athletic comparison group with substantially lower expected exposure to direct head impacts. Biomarker levels remained generally stable in the cross-country cohort, whereas subsets of combat and collision-sport athletes exhibited longitudinal changes in ODE-associated biomarkers, including individuals without clinically recognized concussion or neurological symptoms. Individual head-impact burden and relevant clinical confounders were not systematically quantified, precluding establishment of an exposure–response relationship.

**Conclusion:**

Surface-associated biomarkers on circulating ODEs demonstrated measurable longitudinal changes in subsets of athletes with expected exposure to RHI. These exploratory findings support prospective studies combining ODE-associated biomarker measurements with quantitative head-impact monitoring, clinical characterization, neuroimaging, and neurological outcomes to determine their biological and clinical significance.

## Introduction

Historically, many diseases were initially recognized and diagnosed based on clinical symptoms and physical findings. As their underlying mechanisms were progressively elucidated, biomarkers were identified that enabled more objective diagnosis, risk assessment, and therapeutic monitoring and facilitated the development of targeted interventions. In some conditions, biomarkers have also allowed the identification of asymptomatic individuals at increased risk, creating opportunities for early intervention and prevention. Common examples include lipid profiles for cardiovascular risk assessment (1), glycated hemoglobin (HbA1c) for diabetes screening and management (2), and population-based cancer screening tests that detect disease before symptom onset (3). These precedents illustrate how biomarker-based approaches can complement symptom-based assessments and expand opportunities for preventive medicine.

In contrast, the clinical evaluation of repetitive head impacts (RHI) and concussion remains largely symptom-driven. However, accumulating evidence suggests that biological responses to head impacts may occur in the absence of overt symptoms and may precede clinically recognizable neurological dysfunction (4, 5). Biomarkers capable of detecting and monitoring such subclinical biological responses could therefore provide valuable tools for investigating the cumulative effects of RHI and, ultimately, for longitudinal surveillance and risk assessment.

Conventional imaging modalities such as computed tomography (CT) and magnetic resonance imaging (MRI) are frequently unrevealing following RHI and mild concussion (6). Moreover, asymptomatic athletes rarely undergo such imaging evaluations. Clinically available blood biomarkers, including ubiquitin C-terminal hydrolase L1 (UCH-L1) and glial fibrillary acidic protein (GFAP) (7), have achieved important clinical translation for the acute evaluation of suspected mild traumatic brain injury (TBI). The FDA-cleared Abbott i-STAT TBI assay measures GFAP and UCH-L1 during the acute post-injury period to assist, together with clinical assessment, in determining the need for head CT. UCH-L1 and GFAP are predominantly intracellular proteins associated with neurons and astrocytes, respectively, and their circulating concentrations increase following cellular injury. Because these biomarkers are primarily intended for acute TBI assessment and their concentrations change substantially over the hours and days following injury, their established clinical application differs from the longitudinal assessment of biological responses to repeated, potentially subconcussive head impacts.

Our rationale focuses on the particular vulnerability of axons and myelinated white matter to mechanical forces. Rapid acceleration, deceleration, and rotational forces can produce stretching, shearing, and other mechanical deformation of axons, potentially initiating biological responses even when symptoms are mild or absent and conventional neuroimaging findings are unrevealing (8–11). These responses may include blood–brain barrier disruption, ionic and metabolic disturbances, neurotransmitter dysregulation, neuroinflammation, and accumulation of misfolded proteins (8–11). Oligodendrocytes, which generate and maintain myelin and provide metabolic and trophic support to axons, are closely associated with axonal biology and release extracellular vesicles (EVs) into the local extracellular environment (12). We hypothesized that circulating oligodendrocyte-derived extracellular vesicles (ODEs) may provide a means of interrogating oligodendrocyte- and white-matter-associated biological responses to head impacts. In particular, extracellular molecules generated within the local tissue environment may associate with the surface of ODEs and potentially be detected after these vesicles enter the peripheral circulation (Figure 1). Thus, the intended application of the ODE-based approach investigated here differs from, and could potentially complement, acute TBI biomarkers such as GFAP and UCH-L1: rather than identifying acute intracranial injury, we investigated whether ODE-associated surface markers could provide longitudinal molecular information related to oligodendrocyte and white-matter responses following repeated head-impact exposure.

**Figure 1:**
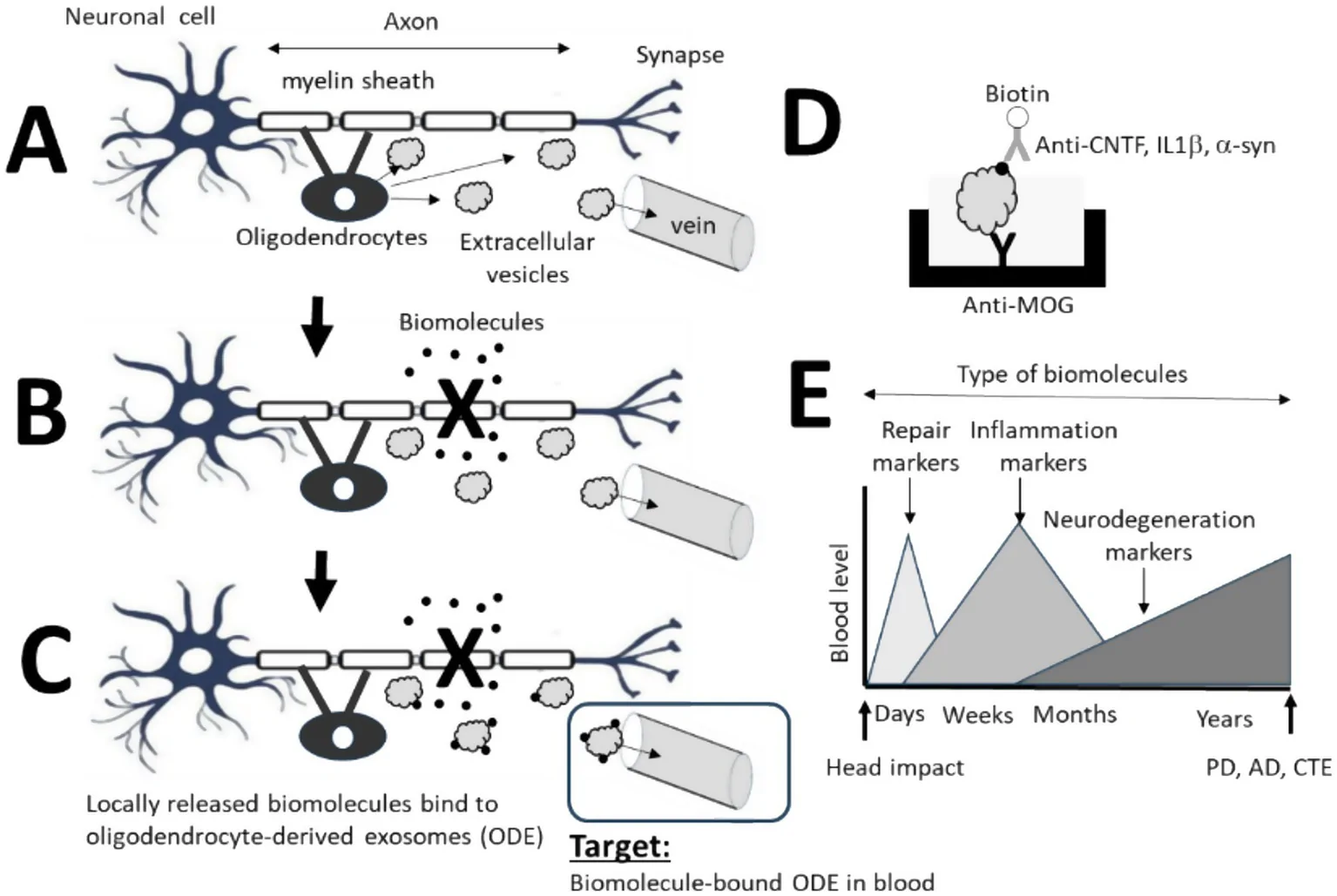
Rationale and assay principle. **(A)** Model of a projection neuron. Axons are ensheathed by myelin maintained by oligodendrocytes, which release extracellular vesicles (EVs), referred to as oligodendrocyte-derived EVs (ODEs), that can enter the circulation. **(B)** Axonal stress. Following axonal stress or injury (marked X), neurons and glial cells release various biomolecules (●) into the local microenvironment. **(C)** Proposed assay target. Biomolecules released in the vicinity of axons may associate with nearby ODEs and subsequently enter the bloodstream. **(D)** Assay principle. A sandwich immunoassay employing anti-myelin oligodendrocyte glycoprotein (MOG) capture antibodies and biotinylated biomolecule-specific probes is performed without prior EV isolation. **(E)** Candidate biomarkers examined in this study. Neurotrophic factors (including CNTF), inflammatory cytokines (including IL1β), and neurotoxic proteins (including α-syn) were selected as associated with repair, inflammation, and neurodegeneration, respectively. PD, Parkinson’s disease; AD, Alzheimer’s disease; CTE, chronic traumatic encephalopathy.

The objective of the present exploratory study was not to establish a standalone diagnostic test or clinical thresholds, but rather to identify candidate ODE-associated blood biomarkers that may change in association with RHI exposure, including in athletes without clinically recognized concussion. Establishing such exposure-associated molecular signals represents an initial step toward prospective longitudinal studies designed to define their temporal dynamics, biological mechanisms, relationships with objectively measured head-impact burden, and associations with clinical outcomes. Importantly, measurable biomarker changes in asymptomatic individuals should not be interpreted as evidence of neurological disease or injury. Rather, such changes may provide an objective means of investigating biological responses to head-impact exposure, including impacts that do not result in clinically recognized concussion (13, 14). Ultimately, integration of validated biomarkers with quantitative head-impact measurements, clinical assessments, and neuroimaging may provide a more comprehensive framework for investigating the short- and long-term consequences of RHI.

## Materials and methods

### Assay principle

Oligodendrocyte-derived extracellular vesicles (ODEs), which are present within the white matter microenvironment and enter the circulation, may reflect local biological responses associated with axonal stress (Figures 1A–C) (12). Plasma ODEs were captured using an anti-myelin oligodendrocyte glycoprotein (MOG) antibody immobilized on a solid support, followed by probe reactions targeting ciliary neurotrophic factor (CNTF), interleukin-1β (IL-1β), and α-synuclein (α-syn) (Figure 1D). These biomarkers were selected to represent neural recovery, neuroinflammation, and neurodegeneration, respectively (Figure 1E). Unlike conventional extracellular vesicle (EV) approaches focused on intravesicular cargo, the present assay measures surface-associated biomolecules while preserving vesicle integrity, thereby providing information on the neural microenvironment.

This exploratory longitudinal study incorporated samples from multiple athletic cohorts and was intended to establish a foundation for subsequent prospective, statistically powered studies.

### Reagents

All reagents were identical to those described in our previous publication (12), except for the additional antibodies used to detect CNTF, IL-1β, and α-synuclein. These included a mouse monoclonal antibody against ciliary neurotrophic factor (CNTF; clone 19, MyBioSource, San Diego, CA), a biotinylated mouse monoclonal antibody against interleukin-1β (IL-1β; clone H1B-98, BioLegend, San Diego, CA), and a mouse monoclonal antibody against α-synuclein (clone 211, Thermo Fisher Scientific). These commercially sourced antibodies have been validated for relevant immunoassay applications according to the manufacturers’ specifications. The anti-CNTF and anti-α-synuclein antibodies were biotinylated using EZ-Link Sulfo-NHS-LC-Biotin (Thermo Fisher Scientific), followed by spin-column purification to remove unbound biotin.

### Assay protocol

The assay was a chemiluminescent sandwich ELISA employing specific capture and detection antibodies (Figure 1D), as previously described (12). Biotinylated detection antibodies were incubated with streptavidin–horseradish peroxidase (HRP), followed by reaction with the chemiluminescent substrate SuperSignal™ (Thermo Fisher Scientific), and relative light units (RLUs) were measured. Captured EVs remained intact, without lysis or permeabilization, thereby preserving their native structure and surface-associated biomolecules. An internal reference plasma sample was assigned a value of 100 U/mL, and RLUs were converted to arbitrary units (U/mL) using four-parameter logistic standard curves (15).

### Scanning electron microscopy

Because SEM imaging requires a flat surface, the bottoms of ELISA wells were excised using a punch, and assays were performed in a humidity chamber to prevent evaporation, similar to our previous studies (12). Samples were sputter-coated with approximately 4 nm of iridium and imaged using a Tescan GAIA3 SEM (Tescan, Czech Republic) at the UC Irvine Materials Research Institute (IMRI).

### Validation of MOG-based ODE capture

The MOG-based ODE capture system used in the present study was previously characterized and validated (12). Captured EVs were visualized by SEM at the bottom of ELISA wells, and the size distribution of eluted ODEs was confirmed by nanoparticle tracking analysis (12). The presence of MOG protein in isolated vesicles was verified by Western blotting, and assay conditions were optimized using the common EV marker CD9 (12). Detailed characterization of the assay platform has been reported previously (12), and the present study was performed using these established methods.

### Plasma samples

#### Control group

Healthy adult plasma samples were purchased from three commercial sources, Innovative Research (Novi, MI), BioIVT (Westbury, NY), and Equitech Enterprise (Kerrville, TX). Commercial plasma samples were used exclusively during assay development and analytical validation to establish assay performance characteristics, including optimization of assay conditions, specificity, and reproducibility. These samples were not used as biological controls in the subsequent clinical analyses.

#### Professional boxers and mixed martial arts fighters (prospective study)

Following approval by the California State Athletic Commission (CSAC) and the Pearl Institutional Review Board (Indianapolis, IN; protocol #2024-0208, approved May 30, 2024), 18 male professional boxers and MMA fighters participating in events organized by SOCA Fights (Fountain Valley, CA) were prospectively enrolled. Written informed consent was obtained from all participants during weigh-ins, one day before competition, when baseline blood samples were collected. Follow-up samples were obtained the day after the bout and at 1, 2, and 5 weeks post-bout.

Capillary blood was collected using the Tasso device (Seattle, WA), and plasma was isolated on the day of collection and stored at −80 °C until analysis. At each sampling time point, participants completed a symptom questionnaire (Supplementary materials). Fight videos were reviewed to document bout outcomes and head-impact exposure. These samples were previously analyzed in a separate study (16).

Before the study, baseline demographic and fight-history information was collected from each fighter, including: (1) age, (2) sex, (3) ethnicity, (4) age or date at which participation in combat sports began, (5) total number of previous fights, (6) number of fights during the preceding year, (7) overall fight record (wins, losses, and draws), (8) fight record during the preceding year, (9) total number of losses by knockout (KO) or technical knockout (TKO), (10) number of losses by KO or TKO during the preceding year, (11) history and timing of hospital visits following a fight, and (12) duration of symptoms following the most recent fight. At the official weigh-in, each fighter underwent a medical examination by an authorized ringside physician and was medically cleared to participate in the scheduled fight. Fighters were also examined by ringside physicians after the fight; none of the participants in this study was referred for hospital evaluation or care. In accordance with CSAC guidelines, fighters who lost by KO or TKO were restricted from sparring for 30-90 days post-fight.

#### High school athletes (retrospective study)

Archived frozen plasma samples from 34 male American football players at two high schools were analyzed. They were all starters, and no player had a severe injury that prevented them to participate in the season. As a non-contact control cohort, high school cross-country runners (*n* = 68) were also analyzed. Their underline health condition was cleared by the school and have never participated in football or other organized contact sports. In football, blood samples were collected monthly from pre-season (July) through post-season (November) using the Tasso Kit. In cross-country starting with 68 runners in July, August, September, and October are collected 5, 5, and 4 athletes out of 68, and we collected 67 in November. All protocols were approved by the Indiana University Institutional Review Board (Bloomington, IN, protocol#1904461516, approved May 02, 2019), and these samples were previously analyzed in another study (16). The consent form for participation was distributed to all participants and signed.

#### College rugby players (retrospective study)

Archived frozen plasma samples from 14 male college rugby players were included. Although we could not identify starters or bench players, all participated in the practices and games throughout the season after the clearance of health status. Blood was collected by venipuncture, with 138 plasma samples obtained three to four times per year, irrespective of concussion history. Ethical approval was granted by the Keio University Ethics Committee (protocol #2022-04, approved July 20, 2022) (Kanagawa, Japan). These samples were also analyzed in a prior study (16). The consent form for participation was distributed to all participants and signed.

Because this project focused on assay development, all available human samples were included regardless of gender, ethnicity, age, or health background.

## Results

### Validation of probes

#### Standard plasma dilution and control results

Serial dilution of a standard plasma sample was used to assess assay performance for CNTF, IL-1β, and α-syn (Figure 2A, left). All analytes showed concentration-dependent signal responses. CNTF and IL-1β exhibited partial signal plateauing at higher concentrations, whereas α-syn maintained a broader linear range across the tested dilutions. Control plasma samples demonstrated inter-individual variability in biomarker levels (Figure 2A, right).

**Figure 2:**
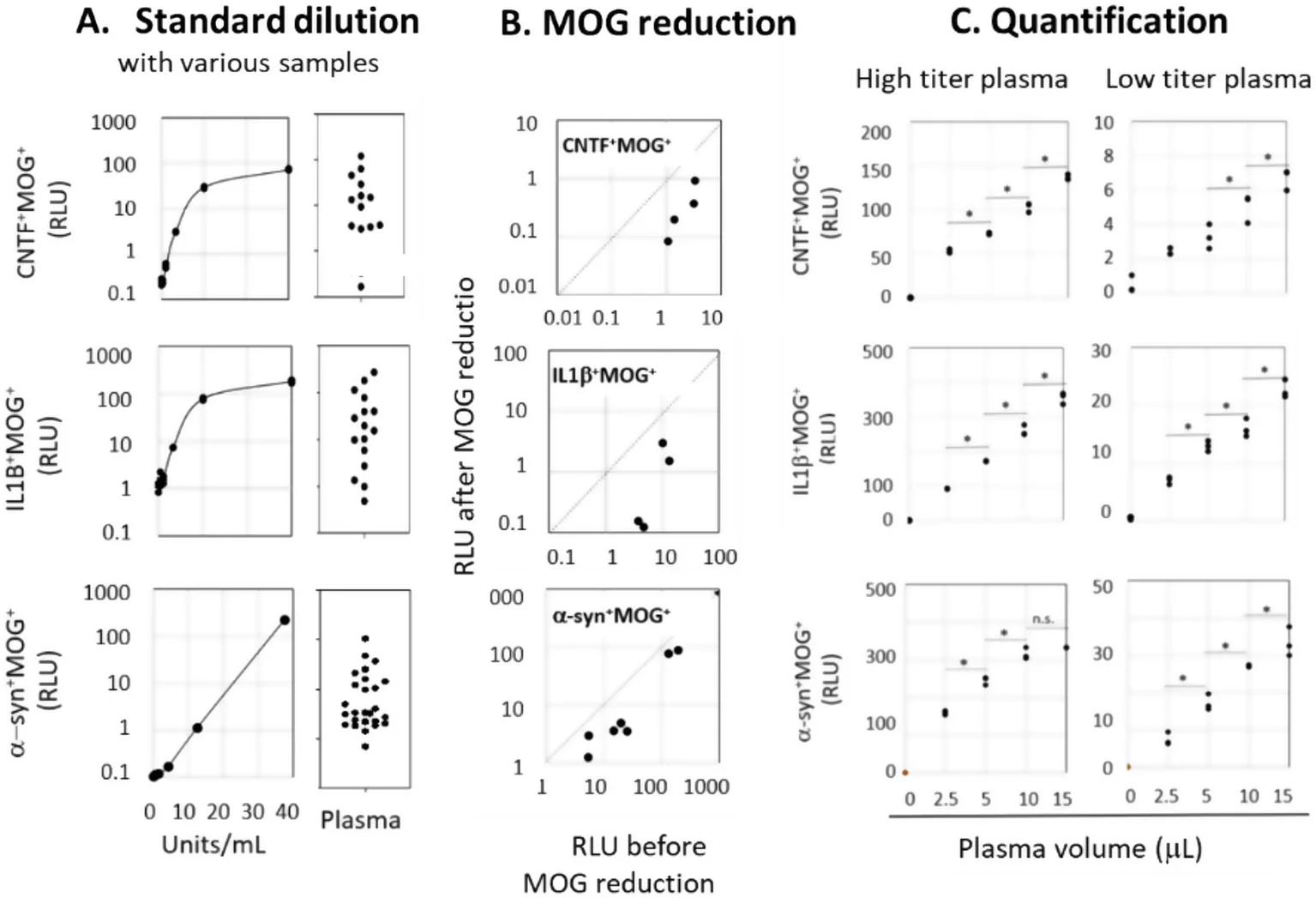
Validation of biomarker probes. **(A)** Standard plasma dilution and control plasma samples. Left: An internal reference plasma sample was assigned a concentration of 100 U/mL and serially diluted for CNTF (top), IL-1β (middle), and α-syn (bottom). The y-axis represents relative light units (RLU) on a logarithmic scale. Right: Measurements from control plasma samples are shown on the same scale as the standard plasma dilution curves (*n* = 16 for CNTF^+^MOG^+^ and IL-1β^+^MOG^+^, *n* = 26 for α-syn^+^MOG^+^). **(B)** MOG depletion. Plasma samples were subjected to MOG^+^ODE depletion as described in Materials and Methods. The x-axis represents RLU before depletion and the y-axis represents RLU after depletion for CNTF^+^MOG^+^ (top), IL-1β^+^MOG^+^ (middle), and α-syn^+^MOG^+^ (bottom). **(C)** Quantification and assay reproducibility. High-titer (left) and low-titer (right) plasma samples were applied at volumes of 0, 2.5, 5, 10, and 15 μL in triplicate and adjusted to a final volume of 40 μL. Captured ODEs were probed with antibodies against CNTF (top), IL-1β (middle), and α-syn (bottom). In each volume, 3 data points were included, although many were overwrapped. *Indicates *p* < 0.05 using unpaired *t*-test between triplicate samples.

#### MOG depletion analysis

To assess association of biomarkers with MOG-positive ODEs, plasma samples were first incubated in anti-MOG–coated wells to capture MOG^+^ODEs. Supernatants were subsequently transferred to a second set of anti-MOG–coated wells and probed for CNTF (Figure 2B, top), IL-1β (Figure 2B, middle), or α-syn (Figure 2B, bottom). Following MOG depletion, signal intensities for CNTF^+^MOG^+^, IL-1β^+^MOG^+^, and α-syn^+^MOG^+^ fractions were reduced compared with pre-depletion measurements.

#### Assay reproducibility and volume dependence

Plasma samples with high and low biomarker levels were applied to anti-MOG–coated wells at volumes of 0, 2.5, 5, 10, and 15 μL in triplicate (final reaction volume 40 μL). Samples were analyzed for CNTF (Figure 2C, top), IL-1β (Figure 2C, middle), or α-syn (Figure 2C, bottom). Signal intensity increased with increasing plasma volume, and replicate measurements showed low variability across conditions.

### Visualization of ODE-bound probes by SEM

Captured EVs were visualized by SEM following labeling with streptavidin-conjugated 20 nm gold nanoparticles. As shown in Figure 3, spherical vesicles measuring approximately 100–200 nm in diameter were observed, consistent with EVs. In our previous study (14), these vesicles were shown to express both CD9 and MOG using gold nanoparticle-labeled antibodies. Negative-control experiments performed without primary antibodies showed no gold nanoparticle labeling, supporting the specificity of the immunolabeling. In the present study, captured ODEs were incubated with biotinylated antibodies against CNTF (Figure 3, top left), IL-1β (Figure 3, top right), or α-syn (Figure 3, bottom), followed by labeling with streptavidin-conjugated gold nanoparticles. Discrete electron-dense signals were observed on the surfaces of a subset of vesicles, consistent with the presence of the corresponding biomarkers. Representative images are shown in Figure 3. These studies were qualitative and were performed to visualize biomarker-associated ODEs rather than to establish definitive membrane topology. However, because the captured EVs were maintained intact and were not permeabilized or lysed before antibody labeling, the detected epitopes were accessible from the external environment and are therefore operationally defined here as EV surface-associated.

**Figure 3:**
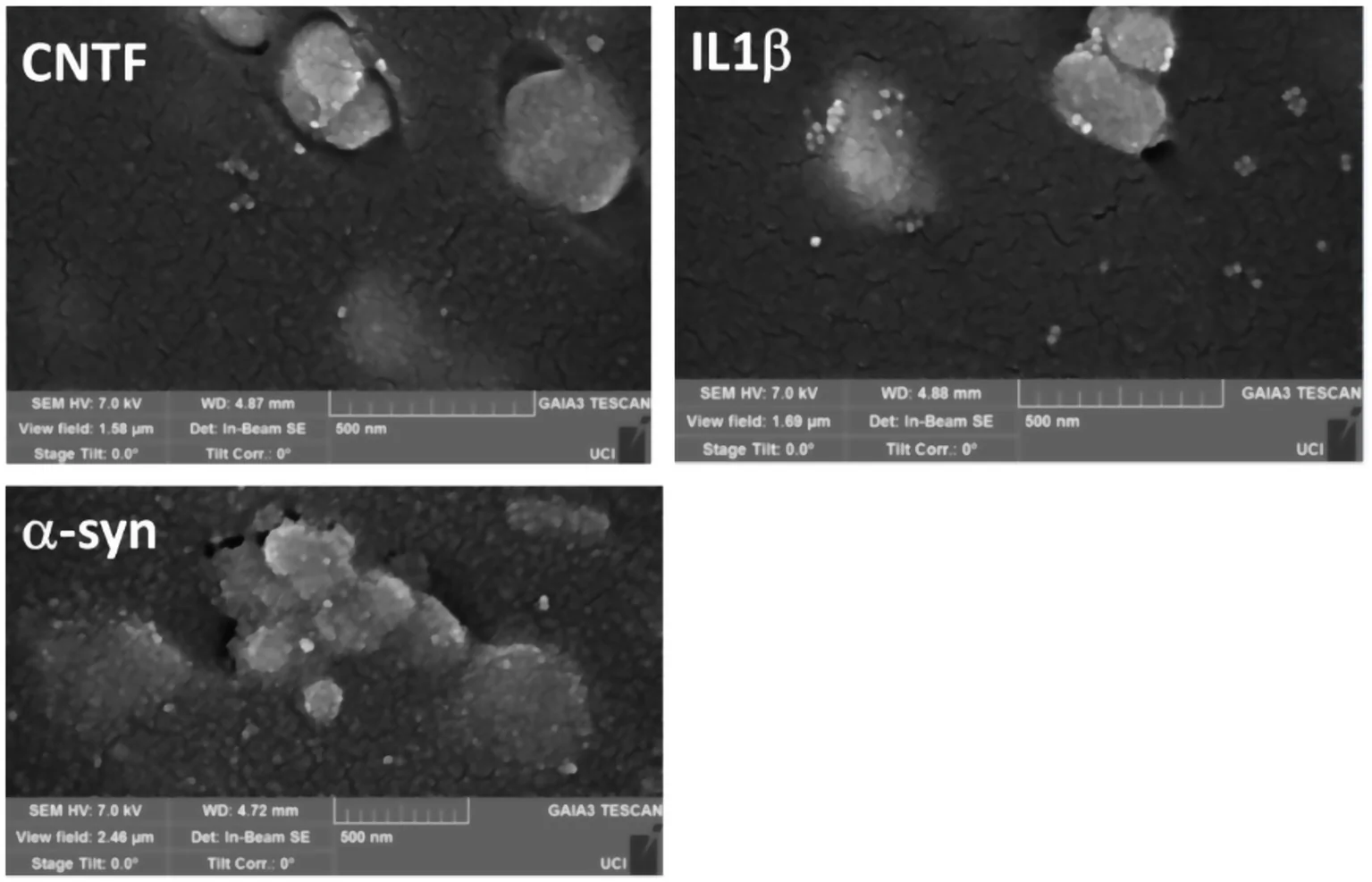
Visualization of biomarker-associated ODEs by scanning electron microscopy (SEM). Captured ODEs immobilized on anti-MOG-coated wells were incubated with biotinylated antibodies against CNTF (top left), IL-1β (top right), or α-syn (bottom), followed by labeling with streptavidin-conjugated 20 nm gold nanoparticles. Representative SEM images show spherical EVs measuring approximately 100–200 nm in diameter. Electron-dense signals (bright white spots) correspond to gold nanoparticles associated with biomarker-positive ODEs. Scale bars are shown in each panel.

### Longitudinal observations in athlete cohorts

The purpose of this section was to illustrate the longitudinal behavior of ODE-associated biomarkers in athletes exposed to RHI. Representative examples are shown for CNTF, IL-1β, and α-syn. These findings are intended to demonstrate methodological feasibility and are not intended for clinical interpretation.

#### CNTF measurements in boxing and MMA

Among the 18 fighters studied, five lost by knockout (KO). Two boxing KO involved direct head impacts and were followed by transient concussion symptoms, whereas the remaining KO resulted from body blows or submission techniques. As shown in Figure 4A, the two fighters who sustained head-punch KO exhibited increased CNTF^+^MOG^+^ levels approximately 1–2 weeks after the bout (▲●), followed by a return toward baseline by week 5. CNTF^+^MOG^+^ levels remained relatively stable in the remaining fighters (○). We could not identify clear correlation to previous Fight history.

**Figure 4:**
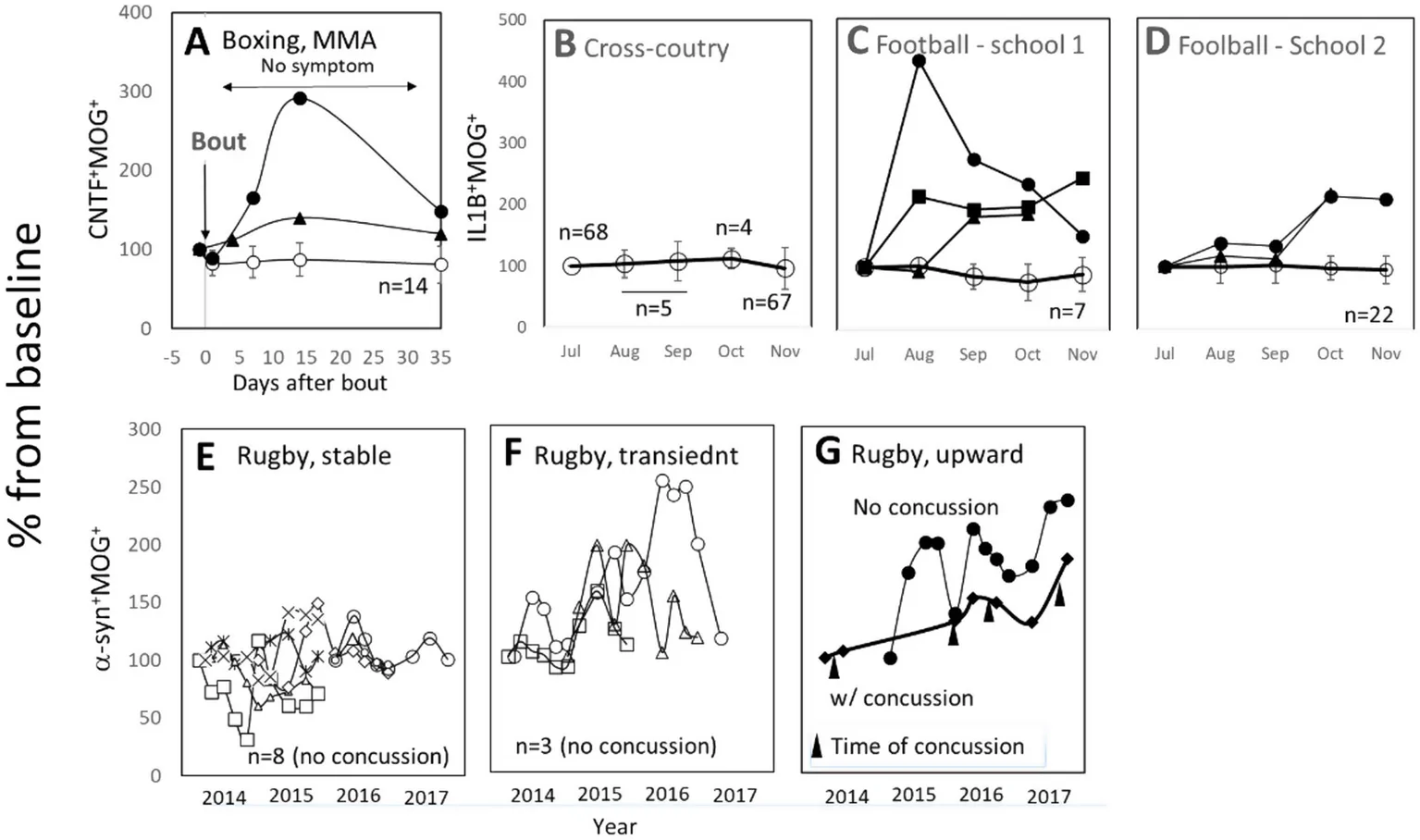
Longitudinal biomarker changes in athlete cohorts following repetitive head impacts. **(A)** CNTF^+^MOG^+^ in boxers and MMA fighters (*n* = 18; 2 below detection limit). Symbols (○) and bars represent mean ± SD of stable subjects (*n* = 14). Y-axis represents percent change from baseline (weigh-in = 100%). X-axis represents days relative to bout. Two fighters who sustained knockout from head impacts and reported transient concussion symptoms (headache, dizziness, sleep disturbance) exhibited increases in CNTF^+^MOG^+^ approximately 1–2 weeks post-bout (▲●), with return toward baseline by 5 weeks. **(B)** IL-1β^+^MOG^+^ in cross-country runners (*n* = 68). This is the non-contact control cohort, starting with 68 runners. August, September, and October are collected 5, 5, and 4 athletes out of 68, and we collected 67 in November. Y-axis represents percent change from baseline (first collection). **(C,D)** IL1β^+^MOG^+^ in high school American football players (*n* = 10 in **C**, *n* = 24 in **D**). Symbols (●) indicate athletes with >200% increase from baseline. Symbols (○) and bars represent mean ± SD of remaining subjects (*n* = 7 in **C**; *n* = 22 in **D**). Blood samples were collected monthly from July to November. Y-axis represents percent change from baseline (first collection). **(E–G)** α-SYN^+^MOG^+^ in college rugby players (*n* = 14). **(E)** Players with stable α-SYN^+^MOG^+^ levels. **(F)** Players showing transient increases during the 4-year observation period (>150% increase from baseline). **(G)** Players showing sustained increases over time; one athlete remained asymptomatic (●), and one experienced four concussions (▲).

#### IL1β measurement in American football

IL1β^+^MOG^+^ levels remained relatively stable in cross-country runners, who served as non-contact controls (Figure 4B). Because of differences in athletic schedules, data from two high schools are shown separately (Figures 4C,D). Most football players exhibited stable IL1β^+^MOG^+^ levels, whereas three players from one school and two players from the second school demonstrated elevations exceeding 200% of preseason values. Some athletes exhibited transient increases, whereas others showed progressive elevations over the course of the season. No clinically recognized concussions were reported in these athletes.

#### α-syn measurement in college rugby players

Longitudinal analysis of α-syn^+^MOG^+^ levels in 14 rugby players over 4 years revealed stable values in most athletes (8/14) (Figure 4E). Three players exhibited transient elevations that subsequently returned to baseline (Figure 4F), whereas two players showed persistent elevations throughout the observation period (Figure 4G). One of these players reported no history of concussion, while the other experienced four concussions during the study period (▲). In all three cohorts, biomarker elevations were not necessarily permanent and, in several individuals, returned to baseline over time.

## Discussion

The athletic cohorts included in this study originated from independent exploratory investigations conducted over multiple years during the progressive development of the analytical platform. Consequently, different biomarker panels were evaluated in different cohorts according to assay availability and the specific biological hypotheses being investigated at the time. The present study was therefore not designed as a unified prospective investigation of RHI, nor was it intended to directly compare biomarker performance across sports or populations. Rather, these cohorts collectively provide an exploratory assessment of the feasibility of quantifying multiple biologically relevant surface-associated markers on circulating ODEs in athletes with differing patterns of expected head-impact exposure using a common analytical approach. Accordingly, differences observed among cohorts should be interpreted as hypothesis-generating observations rather than evidence of comparative biomarker performance or sport-specific effects. Specifically, CNTF was evaluated in the boxing/MMA cohort to explore oligodendrocyte-associated responses and recovery following relatively acute head-impact events. IL-1β was evaluated in American football players to investigate neuroinflammatory responses associated with repetitive head impacts over the course of a season. α-Synuclein was evaluated in the longitudinal collegiate rugby cohort to explore potential longer-term neurodegenerative responses associated with repeated exposure over multiple years. Thus, the different marker selections reflect the exploratory and sequential nature of these independent studies rather than a systematic comparison of all three biomarkers across all sports.

RHI are increasingly recognized as potential contributors to long-term neurological consequences. However, current assessment frameworks remain largely symptom-driven, and biological responses to mild or subconcussive head impacts may occur in the absence of overt neurological symptoms. Consequently, such responses may remain undetected by conventional clinical assessment. The potential long-term consequences of cumulative RHI are underscored by a recent population-based study of former National Football League (NFL) players. Among players who died between 2016 and 2021, the minimum estimated prevalence of neuropathologically confirmed chronic traumatic encephalopathy (CTE) was 24.5%, although the true prevalence could not be determined because brain tissue was unavailable from most deceased players (17). These findings further emphasize the need for objective biomarkers capable of longitudinally assessing biological responses to repetitive head-impact exposure before the development of clinically apparent neurological disease.

In our previous work, we developed an ODE assay to investigate white matter–related disorders, including intracerebral hemorrhage, ischemic stroke, and chemotherapy-associated cognitive impairment (12). Because total circulating ODE levels were not consistently altered following concussion, we explored an alternative approach based on ODE surface-associated biomarkers. Unlike conventional EV analyses that focus primarily on intravesicular cargo, the present method preserves vesicle integrity and detects biomolecules associated with the external surface of captured ODEs. As shown in Figure 3, the presence of selected surface-associated markers was visualized by SEM. Whereas intravesicular cargo is primarily derived from the cell of origin, EV surfaces can interact with extracellular proteins and other biomolecules within the surrounding microenvironment. We therefore hypothesized that surface-associated molecules on circulating ODEs may provide complementary information regarding extracellular biological processes encountered by these vesicles following their release.

Using this platform, we examined CNTF^+^MOG^+^, IL-1β^+^MOG^+^, and α-syn^+^MOG^+^ ODEs as candidate markers selected to reflect neural repair, neuroinflammation, and neurodegenerative processes, respectively. These biomarkers were analytically characterized (Figures 2, 3) and demonstrated measurable longitudinal changes in subsets of athletes with expected exposure to RHI (Figure 4). Biomarker elevations were not uniformly observed across individuals and, in several cases, returned toward baseline over time (Figure 4). The biological basis of this inter-individual variability cannot be determined from the present study. Differences in head-impact burden, timing and severity of exposure, prior head-injury history, other injuries, and individual biological susceptibility may potentially contribute to heterogeneous biomarker responses; however, these factors were not systematically quantified. Accordingly, the observed variability should be regarded as an important feature requiring further investigation in prospectively characterized cohorts rather than as evidence for or against the biological relevance of individual biomarkers.

Several studies have extended the investigation of established TBI biomarkers to circulating EVs. GFAP and UCH-L1 have been measured in total circulating EV populations following TBI, and their EV-associated concentrations and temporal profiles may differ from those measured directly in plasma (18, 19). In addition, GFAP and UCH-L1 have been measured in GluR2-positive brain-derived EVs following mild TBI, further supporting the potential value of analyzing brain-associated biomarkers within selected EV populations (20). These studies, however, examined total or brain-enriched EV populations rather than oligodendrocyte-derived EVs and primarily evaluated EV-associated cargo after vesicle isolation or lysis. The present approach differs by capturing MOG-positive ODEs and measuring biomarkers associated with the external vesicle surface without lysis. Thus, previous EV studies of GFAP and UCH-L1 provide complementary evidence for EV-based TBI biomarker analysis but do not directly evaluate the ODE surface-associated biomarker strategy investigated here.

Cross-country runners were selected as an athletic comparison group with substantially lower expected exposure to direct head impacts than participants in combat or collision sports. However, they should not be considered physiologically inactive or biologically neutral controls, as endurance training may influence systemic inflammation, cardiovascular physiology, metabolism, blood–brain barrier function, and potentially EV release. None of the cross-country runners participated in football or other organized contact sports during the study period. Nevertheless, differences between the athletic groups cannot be attributed exclusively to head-impact exposure because other physiological and sport-related factors were not systematically controlled.

A major challenge in human studies of RHI is the standardization and quantification of head-impact exposure at the individual level. Unlike controlled animal models, the magnitude, frequency, and interval of head impacts cannot be experimentally standardized, and individual behavioral, physiological, and clinical factors may further influence biological responses. In the present exploratory cohorts, histories of prior concussion or TBI, cumulative participation in contact sports, sparring exposure, playing time and position, recent musculoskeletal injury, neurological and other medical conditions, medication use, and participation in additional sports were not systematically characterized or controlled. Accordingly, the present findings cannot establish an exposure–response relationship between cumulative head-impact burden and ODE-associated biomarker levels. Rather than comparing quantitatively defined exposure groups, the present study demonstrates the feasibility of detecting longitudinal changes in ODE-associated biomarkers within individual athletes. These observations support future prospective studies combining serial ODE measurements with quantitative head-impact monitoring and comprehensive clinical characterization to determine whether within-individual biomarker trajectories are associated with cumulative RHI exposure.

Experimental animal models provide complementary evidence supporting the biological rationale for investigating circulating ODE-associated biomarkers following RHI. Unlike observational studies in athletes, animal models permit controlled definition of injury number, frequency, interval, and severity. In mouse models, repetitive mild TBI (rmTBI) has been associated with oligodendrocyte loss and decreased myelin basic protein expression, supporting the vulnerability of oligodendrocytes and myelin to repeated brain injury (21). Similarly, repeated closed-head injury in developing mice produced long-term abnormalities in oligodendrocyte development and white matter microstructure that persisted for months after injury (22). Even after a single mild TBI, dynamic demyelination followed by remyelination and generation of new oligodendrocytes has been demonstrated in the corpus callosum, indicating that oligodendrocyte and myelin responses evolve over time following brain injury (23). In parallel, experimental TBI has been shown to produce time-dependent alterations in circulating EV abundance, cellular subpopulations, and molecular composition, including changes in EV-associated neuronal, microglial, and astrocytic markers (24). Collectively, these experimental findings establish the biological vulnerability of oligodendrocytes and white matter to traumatic brain injury and support the investigation of circulating ODE-associated molecular signals as candidate longitudinal indicators of these responses. However, whether circulating ODE-associated changes quantitatively reflect the frequency, severity, or cumulative burden of head impacts in humans remains to be established in prospectively characterized studies.

The present study was intended as a methodological and exploratory investigation rather than a clinical validation study. The assay is based on standard ELISA technology, requires only small volumes of venous or capillary blood, and is compatible with archived frozen plasma samples. These practical features may facilitate future longitudinal studies involving larger populations and repeated within-individual measurements. However, the present study does not establish population reference ranges, normal or abnormal thresholds, diagnostic performance, or clinical recommendations. Establishing the clinical validity of these candidate biomarkers will require larger, prospectively characterized cohorts incorporating quantitative head-impact monitoring, relevant clinical and medical histories, neuroimaging, cognitive assessments, and long-term clinical follow-up. Such studies will be necessary to determine whether longitudinal ODE-associated biomarker trajectories are related to cumulative head-impact exposure and subsequent neurological outcomes.

Although the present study focused on ODEs, the analytical concept is not restricted to this EV population. The same strategy could potentially be adapted to other cell-associated EV populations by selecting appropriate capture and surface biomarkers. Surface-associated biomarker analysis may therefore provide information complementary to conventional EV cargo analysis and represents a broader analytical approach for investigating dynamic biological responses in longitudinal studies.

## Data Availability

The raw data supporting the conclusions of this article will be made available by the authors, without undue reservation.

## Glossary

α-syn: α-synuclein
BDNF: brain-derived neurotrophic factor
CNTF: ciliary neurotrophic factor
CSAC: California State Athletic Commission
CT: Computed Tomography
ELISA: enzyme-linked immunosorbent assay
EVs: extracellular vesicles
GDNF: glial cell derived neurotrophic facto
GFAP: glial fibrillary acidic protein
HbA1c: glycated hemoglobin
ICs: inflammatory cytokines
IL1b: interleukin 1b
IMRI: UC Irvine Materials Research Institute
IRB: Institutional Review Board
KO: knockout
MMA: mixed martial arts
MOG: myelin oligodendrocyte glycoprotein
MRI: Magnetic Resonance Imaging
NTFs: neurotrophic factors
ODE: OL-derived exosomes
PD: Parkinson’s disease
PET: positron emission tomography
RHI: Repetitive mild head impacts
RLU: relative light units
SEM: scanning electron microscopy
UCH-L1: ubiquitin C-terminal hydrolase L1
